# Crystal structure and Hirshfeld surface analysis of dimeth­yl(phen­yl)phosphine sulfide

**DOI:** 10.1107/S2056989024005668

**Published:** 2024-06-18

**Authors:** Robin Risken, Yasin Mehmet Kuzu, Annika Schmidt, Carsten Strohmann

**Affiliations:** a Inorganic Chemistry, TU Dortmund University, Otto-Hahn Str. 6, 44227 Dortmund, Germany; University of Aberdeen, United Kingdom

**Keywords:** crystal structure, Hirshfeld surface analysis, phosphine sulfide

## Abstract

The title compound, C_8_H_11_PS, which melts below room temperature, was crystallized at low temperature. The P—S bond length is 1.9623 (5) Å and the major contributors to the Hirshfeld surface are H⋯H (58.1%), S⋯H/H⋯S (13.4%) and C⋯H/H⋯C contacts (11.7%).

## Abstract 2

The title compound, C_8_H_11_PS, which melts below room temperature, was crystallized at low temperature. The P—S bond length is 1.9623 (5) Å and the major contributors to the Hirshfeld surface are H⋯H, S⋯H/H⋯S and C⋯H/H⋯C contacts.

## 1. Chemical context

**Figure 1:**
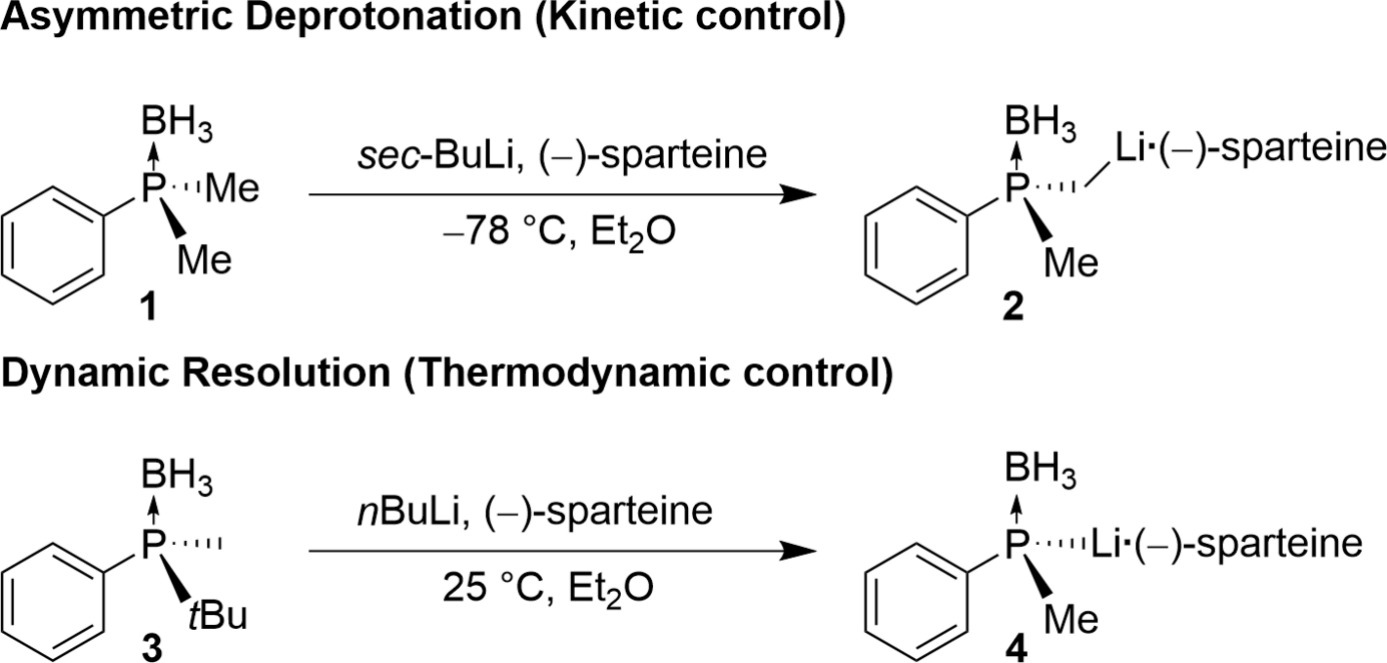
Kinetic and thermodynamic approaches to synthesize chiral phosphine boranes.

**Figure 2:**
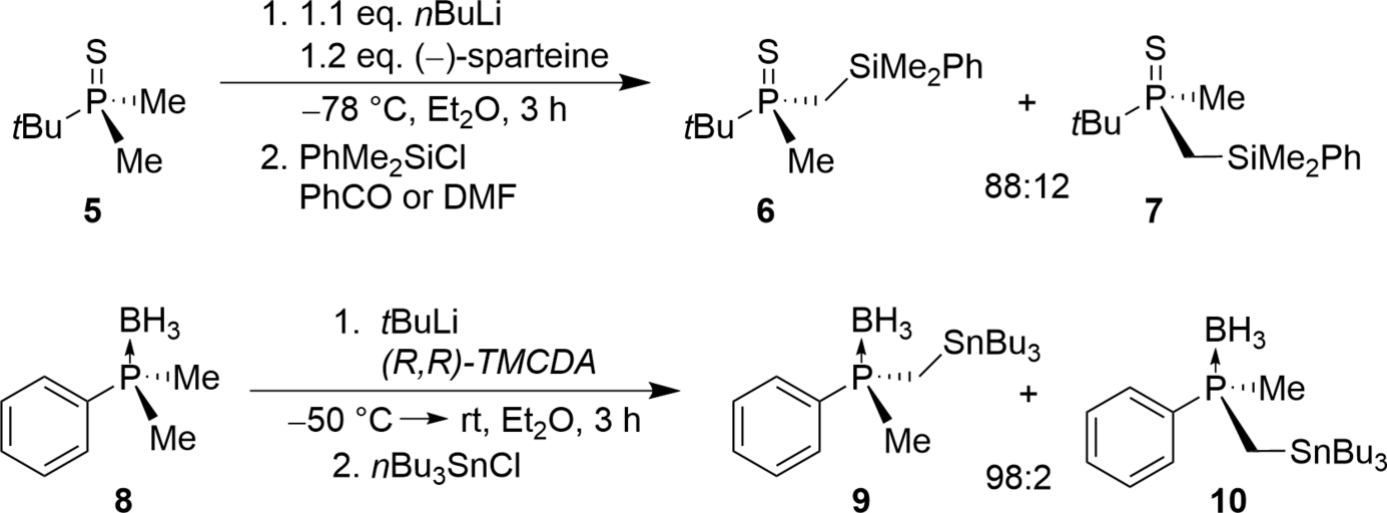
More recent synthetic approaches for chiral phosphine sulfides and boranes.

The structure of the title compound, C_8_H_11_PS, **11**, is inter­esting for two reasons: firstly, the crystals are very temperature sensitive and secondly the chemical background of the substance itself. Although **11** has been known since 1962 (Monsanto Chemicals, 1962 ▸) and even commercially available, no crystal structure has been obtained until now. This might be due to its low melting point, which made measurements very difficult and only feasible with X-Temp 2 (Stalke, 1998 ▸), which makes crystal picking and mounting possible at very low temperatures. Phospho­rus-based mol­ecules are used in a large variety of different chemical applications as chiral ligands for enanti­oselective catalysis (Grabulosa, 2011 ▸). Compound **11** is a prochiral building block for *P*-stereogenic biphosphine ligands, which are used for transition-metal-catalyzed asymmetric reactions (Tang & Zhang, 2003 ▸). This application makes an enanti­oselective synthesis indispensable, which is why different approaches have been reported. For the chemically similar phosphine–boranes, the desired enanti­omer can be synthesized either under kinetic control with *sec*-BuLi and (−)-sparteine *via* an asymmetric deprotonation (Muci *et al.*, 1995 ▸) or a thermodynamically controlled reaction with *n-*BuLi and (−)-sparteine *via* dynamic resolution (Wolfe & Livinghouse, 1998 ▸) (Fig. 1 ▸). For phosphine sulfides like compound **11**, the synthetic approach is quite similar. Using *n*-BuLi and (−)-sparteine in Et_2_O results in an enanti­ometric ratio (*e.r*.) of 88:12 *via* a kinetically controlled reaction (Gammon *et al.*, 2010 ▸) (Fig. 2 ▸). By trapping the li­thia­ted inter­mediate, not only has a higher enanti­oselectivity of *e.r*. = 93:7 been achieved, but it has also been discovered that the enanti­omers can inter­convert at temperatures above 253 K. One of the most recent synthetic approaches for phosphine–boranes relies on the much cheaper (*R*,*R*)-TMCDA instead of (−)-sparteine and crystallization-induced dynamic resolution (CIDR). This synthesis achieves up to 80% yield and enanti­oselectivity of 98:2 (Kuzu *et al.*, 2024 ▸).
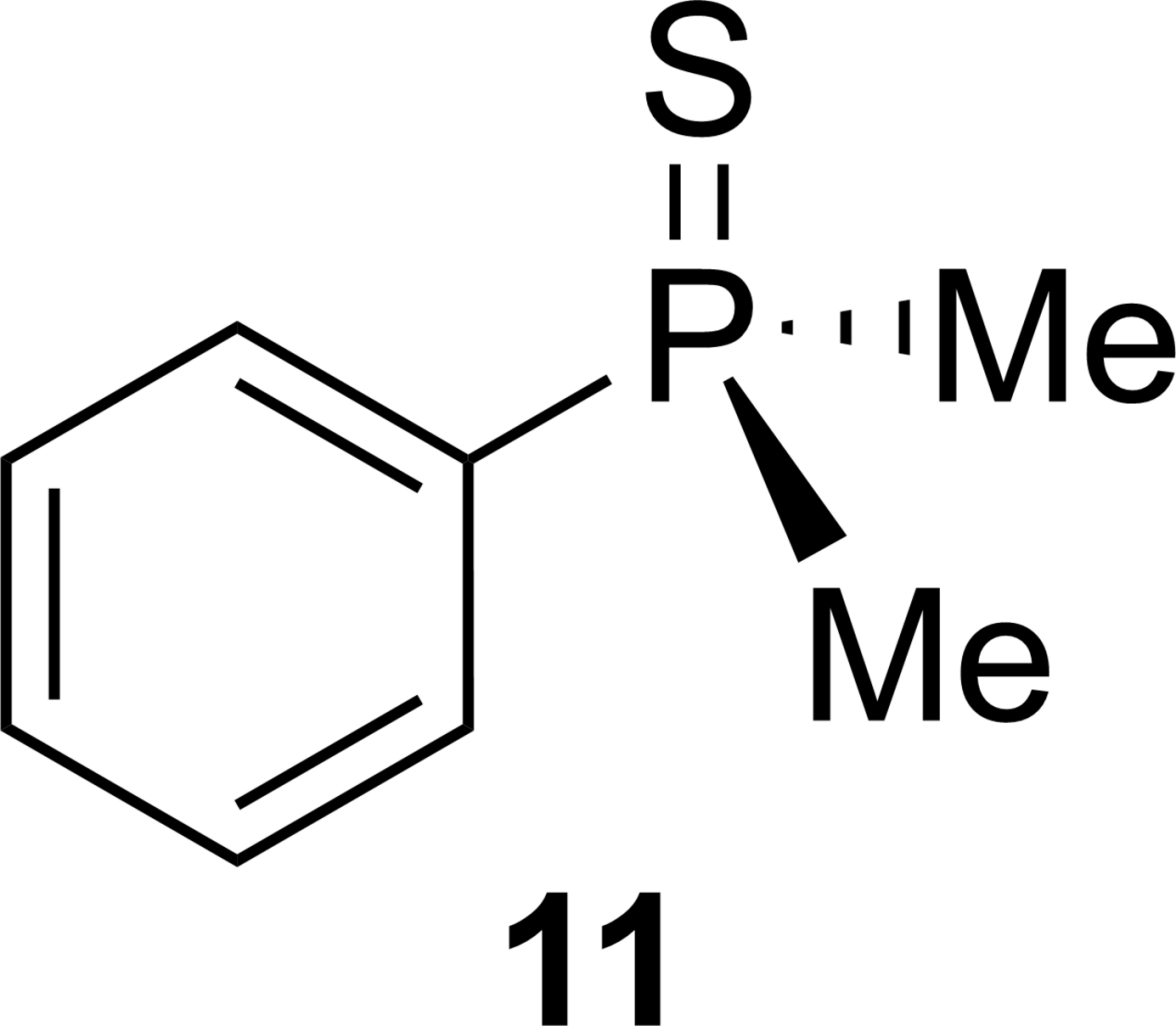


## 2. Structural commentary

Compound **11**, which was crystallized from toluene at 193 K, forms colorless needles in the monoclinic space group *P*2_1_/*n*. The P-tetra­hedral mol­ecule consists of two methyl groups and one phenyl group bound to the phosphine sulfide unit. The P1—S1 bond length of 1.9623 (5) Å matches the typical bond length for this species (Verschoor-Kirss *et al.*, 2016 ▸; Blake *et al.*, 1981 ▸). The phospho­rus bond angles vary from 105.58 (6)° for C1—P1—C3 to 113.55 (5)° for C2—P1—S1. These angles are slightly distorted from the nominal angle of 109.5°, which is probably caused by the different steric effects of the substituents. The bond lengths and angles of the C3–C8 phenyl ring match with the typical lengths and angles for this familiar group (Lide, 2005 ▸). Atom S1 is displaced from the plane of the C3–C8 ring by −0.549 (1) Å with corresponding deviations for atoms C1 and C2 of the methyl groups of 1.839 (2) and −0.781 (1) Å, respectively; the C4—C3—P1—S1 torsion angle is 23.85 (12)°.

## 3. Supra­molecular features

**Figure 3:**
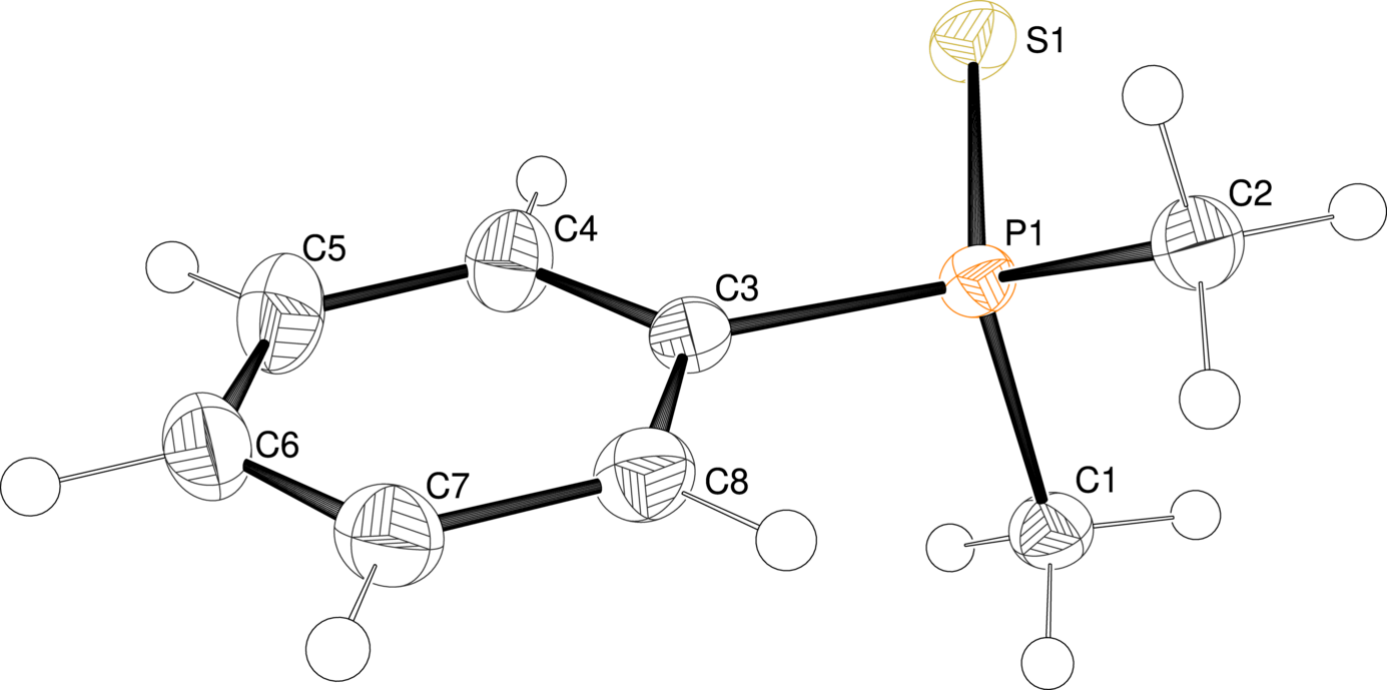
The mol­ecular structure of **11** showing 50% displacement ellipsoids.

**Figure 4:**
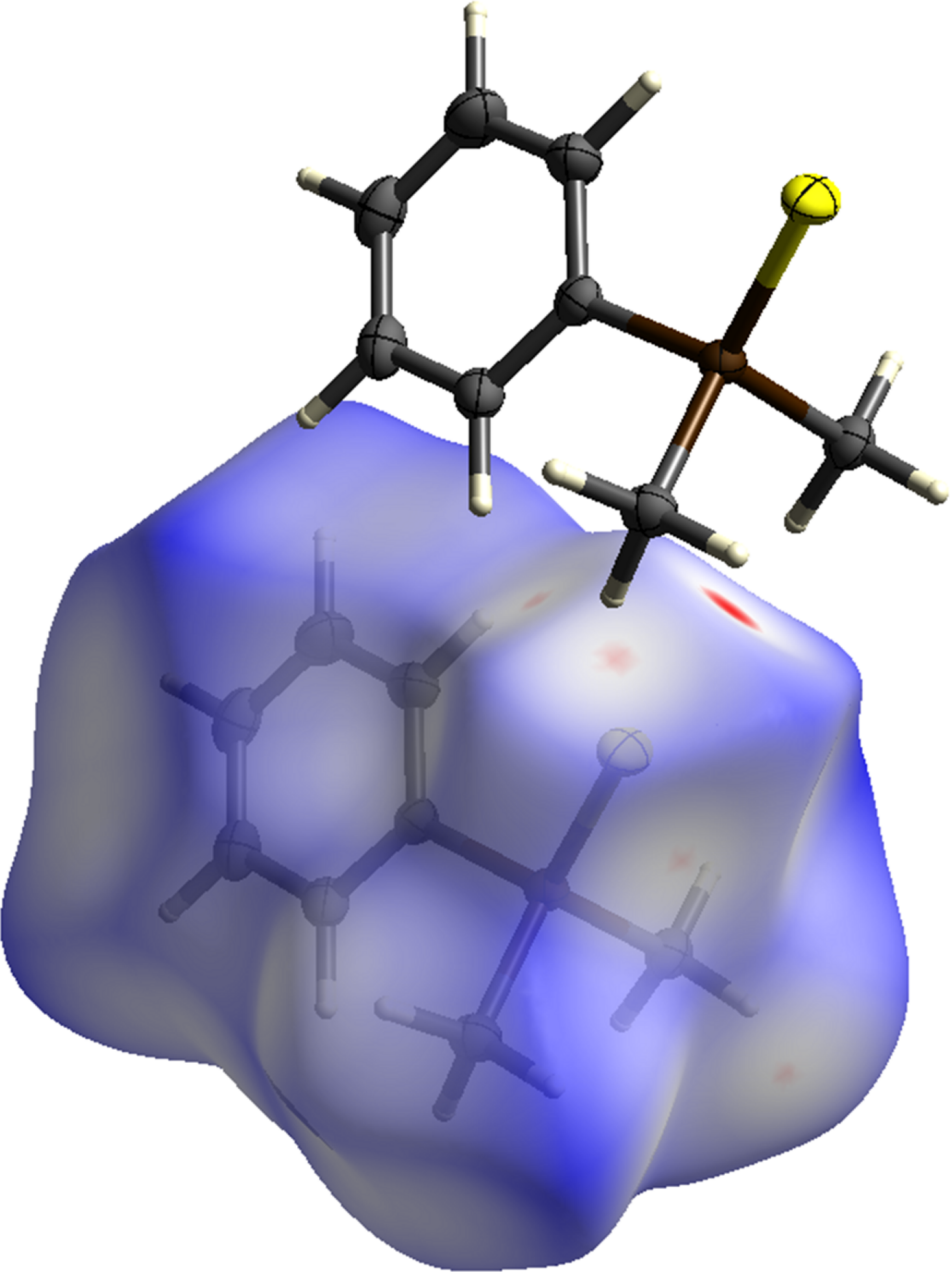
Hirshfeld surface analysis of **11** showing close contacts in the crystal.

**Figure 5:**
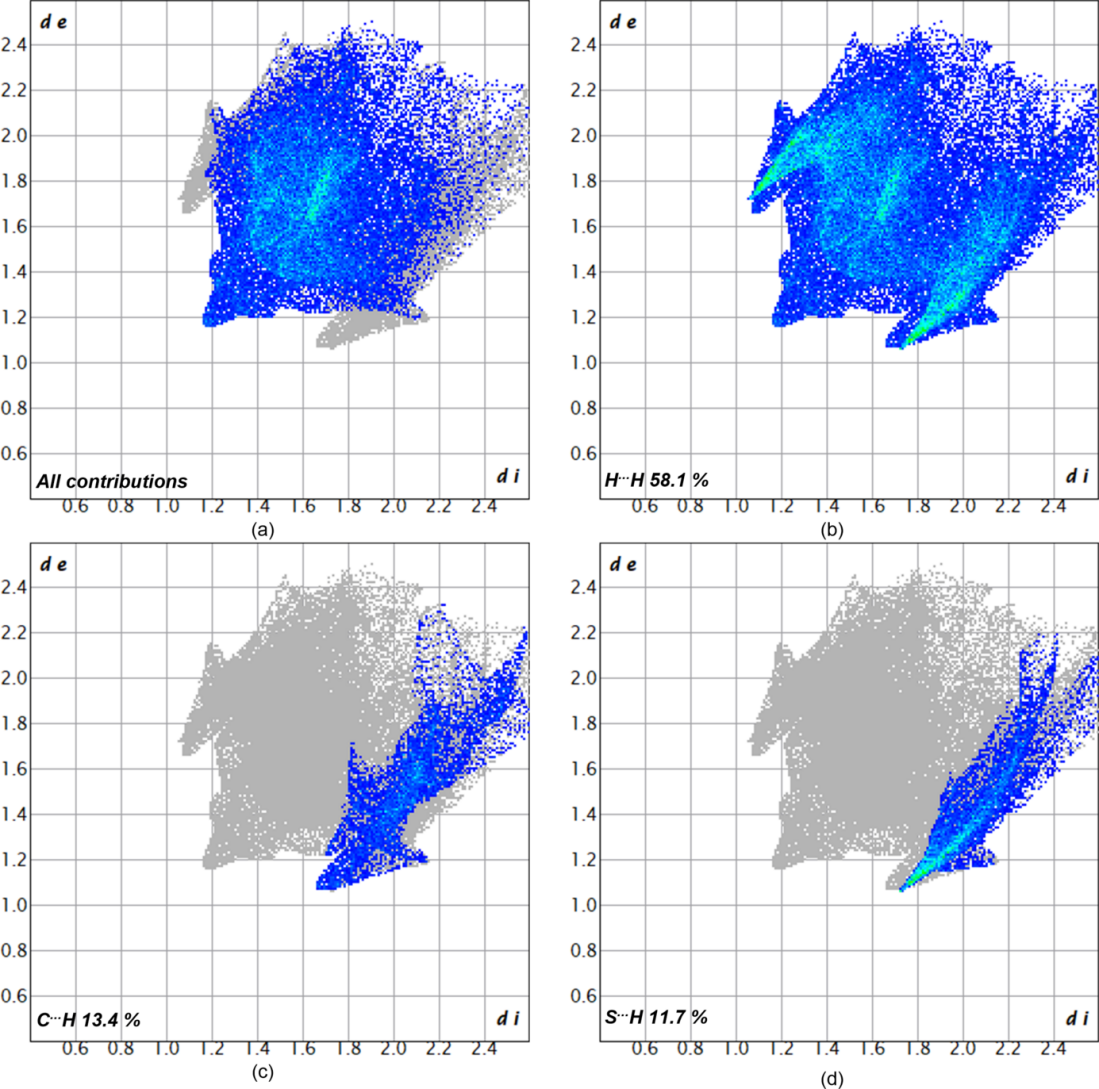
Two-dimensional fingerprint plots for compound **11**, showing (*a*) all contributions and (*b*)–(*d*) contributions between specific inter­acting atom pairs.

To better understand the inter­molecular inter­actions of **11**, a Hirshfeld surface analysis was performed. In Fig. 3 ▸ the Hirshfeld surface analysis is mapped over *d*_norm_ in the range of −0.07 to 1.20 a.u. (Spackman & Jayatilaka, 2009 ▸) and generated by *CrystalExplorer21* (Spackman *et al.*, 2021 ▸) using red dots to represent close contacts. Atom S1 has close contacts to atoms H1*A* (H⋯S = 2.87 Å) and H2*C* (2.95 Å) of the methyl groups of a neighboring mol­ecule displaced by translation in the *a*-axis direction. For further visualization of the percentage of the respective inter­actions, two-dimensional fingerprint plots (McKinnon *et al.*, 2007 ▸) were generated and these are shown in Fig. 4 ▸. The most significant contacts in the solid state are the H⋯H inter­actions, contributing 58.1% of the total surface (Fig. 5 ▸). The S⋯H/H⋯S (13.4%) and C⋯H/H⋯C inter­actions (11.7%) are less impactful in comparison.

## 4. Database survey

Similar mol­ecules to **11** can vary either in the attached heteroatom such as the previously discussed phosphine boranes (Muci *et al.*, 1995 ▸; Kuzu *et al.*, 2024 ▸) or they can vary in their organic substituents (Gammon *et al.*, 2010 ▸). A search in the Cambridge Structural Database (WebCSD, March 2024; Groom *et al.*, 2016 ▸) for phosphine sulfides lead to many similar structures with different organic substituents. Some of those structures contain aromatic substituents such as three phenyl rings (CSD refcode BAQTOC; Arca *et al.*, 1999 ▸) or even larger substituents like an anthracene group (BARWEA; Schillmöller *et al.*, 2021 ▸). Structures with smaller substituents are also known, for example, butyro­nitrile (KADJEE; Blake *et al.*, 1981 ▸).

## 5. Synthesis and crystallization

In a round-bottom flask equipped with a condenser dimeth­yl(phen­yl)phosphane (1.00 g, 7.24 mmol, 1 eq.) and sulfur (2.23 g, 8.69 mmol, 1.2 eq.) were dissolved in 20 ml of toluene. While stirring, the mixture was heated under reflux to 373 K and then stirred overnight without heating. The resulting mixture was filtered through 3 cm of celite and washed with diethyl ether. The organic phase was dried with magnesium sulfate and the solvent was removed *in vacuo*, yielding a slightly yellow oil of dimeth­yl(phen­yl)phosphine sulfide (1.04 g, 85%). The oil was dissolved in hot toluene and recrystallized at 193 K, forming colorless needles.

## 6. Refinement

Crystal data, data collection and structure refinement details are summarized in Table 1 ▸. All H atoms were geometrically placed (C—H = 0.95–0.98 Å) and refined as riding atoms with *U*_iso_(H) = 1.2*U*_eq_(C) or 1.5*U*_eq_(methyl C).

## Supplementary Material

Crystal structure: contains datablock(s) I. DOI: 10.1107/S2056989024005668/hb8100sup1.cif

Structure factors: contains datablock(s) I. DOI: 10.1107/S2056989024005668/hb8100Isup2.hkl

Supporting information file. DOI: 10.1107/S2056989024005668/hb8100Isup3.cml

CCDC reference: 2362349

Additional supporting information:  crystallographic information; 3D view; checkCIF report

## Figures and Tables

**Table 1:** Experimental details

Crystal data	
Chemical formula	C_8_H_11_PS
*M*_r_	170.20
Crystal system, space group	Monoclinic, *P*2_1_/*n*
Temperature (K)	100
*a*, *b*, *c* (Å)	6.2805 (2), 7.6549 (2), 19.3578 (8)
β (°)	99.372 (2)
*V* (Å^3^)	918.23 (5)
*Z*	4
Radiation type	Cu *K*α
μ (mm^−1^)	4.17
Crystal size (mm)	0.14 × 0.13 × 0.10

Data collection	
Diffractometer	Bruker APEXII CCD
Absorption correction	Multi-scan (*SADABS*; Krause *et al.*, 2015 ▸)
*T*_min_, *T*_max_	0.465, 0.587
No. of measured, independent and observed [*I* > 2σ(*I*)] reflections	12606, 1886, 1745
*R*_int_	0.029
(sin θ/λ)_max_ (Å^−1^)	0.626

Refinement	
*R*[*F*^2^ > 2σ(*F*^2^)], *wR*(*F*^2^), *S*	0.024, 0.064, 1.06
No. of reflections	1886
No. of parameters	93
H-atom treatment	H-atom parameters constrained
Δρ_max_, Δρ_min_ (e Å^−3^)	0.34, −0.23

Computer programs: *APEX2* and *SAINT* (Bruker, 2018 ▸), *SHELXT* (Sheldrick, 2015*a* ▸), *SHELXL2014/7* (Sheldrick, 2015*b* ▸) and *OLEX2* (Dolomanov *et al.*, 2009 ▸).

## supplementary crystallographic information

### Dimethyl(phenyl)phosphanethione Crystal data

**Table:**

C_8_H_11_PS	*F*(000) = 360
*M**_r_* = 170.20	*D*_x_ = 1.231 Mg m^−^^3^
Monoclinic, *P*2_1_/*n*	Cu *K*α radiation, λ = 1.54178 Å
*a* = 6.2805 (2) Å	Cell parameters from 3458 reflections
*b* = 7.6549 (2) Å	θ = 4.6–79.2°
*c* = 19.3578 (8) Å	µ = 4.17 mm^−^^1^
β = 99.372 (2)°	*T* = 100 K
*V* = 918.23 (5) Å^3^	Needle, colourless
*Z* = 4	0.14 × 0.13 × 0.10 mm

### Dimethyl(phenyl)phosphanethione Data collection

**Table:**

Bruker APEXII CCD diffractometer	1745 reflections with *I* > 2σ(*I*)
φ and ω scans	*R*_int_ = 0.029
Absorption correction: multi-scan (SADABS; Krause *et al.*, 2015)	θ_max_ = 74.7°, θ_min_ = 4.6°
*T*_min_ = 0.465, *T*_max_ = 0.587	*h* = −7→7
12606 measured reflections	*k* = −9→7
1886 independent reflections	*l* = −24→23

### Dimethyl(phenyl)phosphanethione Refinement

**Table:**

Refinement on *F*^2^	Primary atom site location: dual
Least-squares matrix: full	Hydrogen site location: inferred from neighbouring sites
*R*[*F*^2^ > 2σ(*F*^2^)] = 0.024	H-atom parameters constrained
*wR*(*F*^2^) = 0.064	*w* = 1/[σ^2^(*F*_o_^2^) + (0.031*P*)^2^ + 0.342*P*] where *P* = (*F*_o_^2^ + 2*F*_c_^2^)/3
*S* = 1.06	(Δ/σ)_max_ = 0.001
1886 reflections	Δρ_max_ = 0.34 e Å^−^^3^
93 parameters	Δρ_min_ = −0.23 e Å^−^^3^
0 restraints	

### Dimethyl(phenyl)phosphanethione Special details

**Table:**

Geometry. All esds (except the esd in the dihedral angle between two l.s. planes) are estimated using the full covariance matrix. The cell esds are taken into account individually in the estimation of esds in distances, angles and torsion angles; correlations between esds in cell parameters are only used when they are defined by crystal symmetry. An approximate (isotropic) treatment of cell esds is used for estimating esds involving l.s. planes.

### Dimethyl(phenyl)phosphanethione Fractional atomic coordinates and isotropic or equivalent isotropic displacement parameters (Å^2^)

**Table:**

	*x*	*y*	*z*	*U*_iso_*/*U*_eq_	
P1	0.42807 (5)	0.75930 (4)	0.57224 (2)	0.02071 (10)	
S1	0.73232 (5)	0.79603 (5)	0.56424 (2)	0.02966 (11)	
C3	0.3919 (2)	0.57849 (16)	0.62992 (6)	0.0225 (3)	
C8	0.1928 (2)	0.49493 (18)	0.62617 (7)	0.0280 (3)	
H8	0.0754	0.5285	0.5913	0.034*	
C2	0.2586 (2)	0.71634 (18)	0.48984 (7)	0.0267 (3)	
H2A	0.3014	0.6055	0.4708	0.040*	
H2B	0.2743	0.8110	0.4570	0.040*	
H2C	0.1078	0.7091	0.4968	0.040*	
C1	0.3115 (2)	0.94645 (17)	0.60830 (7)	0.0268 (3)	
H1A	0.1620	0.9205	0.6133	0.040*	
H1B	0.3142	1.0466	0.5769	0.040*	
H1C	0.3950	0.9741	0.6543	0.040*	
C4	0.5618 (2)	0.52745 (19)	0.68113 (7)	0.0320 (3)	
H4	0.6990	0.5816	0.6836	0.038*	
C7	0.1657 (2)	0.36298 (19)	0.67315 (8)	0.0331 (3)	
H7	0.0303	0.3056	0.6700	0.040*	
C6	0.3345 (3)	0.31482 (19)	0.72440 (8)	0.0357 (3)	
H6	0.3152	0.2250	0.7567	0.043*	
C5	0.5320 (3)	0.3975 (2)	0.72876 (8)	0.0398 (4)	
H5	0.6477	0.3654	0.7645	0.048*	

### Dimethyl(phenyl)phosphanethione Atomic displacement parameters (Å^2^)

**Table:**

	*U*^11^	*U*^22^	*U*^33^	*U*^12^	*U*^13^	*U*^23^
P1	0.01838 (17)	0.02128 (17)	0.02246 (17)	0.00095 (11)	0.00330 (12)	0.00012 (12)
S1	0.01915 (17)	0.03201 (19)	0.0385 (2)	0.00062 (12)	0.00678 (13)	0.00507 (14)
C3	0.0253 (6)	0.0205 (6)	0.0223 (6)	0.0025 (5)	0.0058 (5)	−0.0015 (5)
C8	0.0261 (7)	0.0248 (6)	0.0337 (7)	−0.0002 (5)	0.0066 (5)	−0.0004 (5)
C2	0.0266 (7)	0.0291 (7)	0.0238 (6)	0.0010 (5)	0.0021 (5)	−0.0011 (5)
C1	0.0285 (7)	0.0236 (6)	0.0293 (6)	0.0023 (5)	0.0075 (5)	−0.0008 (5)
C4	0.0304 (7)	0.0315 (7)	0.0321 (7)	−0.0014 (6)	−0.0011 (6)	0.0027 (6)
C7	0.0377 (8)	0.0251 (7)	0.0396 (8)	−0.0021 (6)	0.0152 (6)	−0.0005 (6)
C6	0.0553 (10)	0.0250 (7)	0.0300 (7)	0.0032 (6)	0.0165 (7)	0.0035 (6)
C5	0.0478 (9)	0.0368 (8)	0.0319 (7)	0.0039 (7)	−0.0027 (7)	0.0077 (6)

### Dimethyl(phenyl)phosphanethione Geometric parameters (Å, º)

**Table:**

P1—S1	1.9623 (5)	C1—H1A	0.9800
P1—C3	1.8158 (13)	C1—H1B	0.9800
P1—C2	1.7977 (13)	C1—H1C	0.9800
P1—C1	1.8004 (13)	C4—H4	0.9500
C3—C8	1.3957 (19)	C4—C5	1.390 (2)
C3—C4	1.3893 (18)	C7—H7	0.9500
C8—H8	0.9500	C7—C6	1.379 (2)
C8—C7	1.388 (2)	C6—H6	0.9500
C2—H2A	0.9800	C6—C5	1.383 (2)
C2—H2B	0.9800	C5—H5	0.9500
C2—H2C	0.9800		
			
C3—P1—S1	112.26 (4)	P1—C1—H1A	109.5
C2—P1—S1	113.55 (5)	P1—C1—H1B	109.5
C2—P1—C3	106.91 (6)	P1—C1—H1C	109.5
C2—P1—C1	105.68 (6)	H1A—C1—H1B	109.5
C1—P1—S1	112.28 (5)	H1A—C1—H1C	109.5
C1—P1—C3	105.58 (6)	H1B—C1—H1C	109.5
C8—C3—P1	121.21 (10)	C3—C4—H4	119.9
C4—C3—P1	119.64 (10)	C3—C4—C5	120.24 (14)
C4—C3—C8	119.08 (12)	C5—C4—H4	119.9
C3—C8—H8	119.9	C8—C7—H7	119.9
C7—C8—C3	120.25 (13)	C6—C7—C8	120.26 (14)
C7—C8—H8	119.9	C6—C7—H7	119.9
P1—C2—H2A	109.5	C7—C6—H6	120.1
P1—C2—H2B	109.5	C7—C6—C5	119.89 (13)
P1—C2—H2C	109.5	C5—C6—H6	120.1
H2A—C2—H2B	109.5	C4—C5—H5	119.9
H2A—C2—H2C	109.5	C6—C5—C4	120.26 (14)
H2B—C2—H2C	109.5	C6—C5—H5	119.9
			
P1—C3—C8—C7	−176.66 (10)	C8—C7—C6—C5	−0.4 (2)
P1—C3—C4—C5	175.48 (12)	C2—P1—C3—C8	−34.24 (12)
S1—P1—C3—C8	−159.38 (10)	C2—P1—C3—C4	148.99 (11)
S1—P1—C3—C4	23.85 (12)	C1—P1—C3—C8	77.98 (12)
C3—C8—C7—C6	0.8 (2)	C1—P1—C3—C4	−98.79 (12)
C3—C4—C5—C6	1.7 (2)	C4—C3—C8—C7	0.1 (2)
C8—C3—C4—C5	−1.4 (2)	C7—C6—C5—C4	−0.8 (2)

## References

[bb1] Arca, M., Demartin, F., Devillanova, F. A., Garau, A., Isaia, F., Lippolis, V. & Verani, G. (1999). *J. Chem. Soc. Dalton Trans.* pp. 3069–3073.

[bb2] Blake, A. J., Howie, R. A. & McQuillan, G. P. (1981). *Acta Cryst.* B**37**, 1959–1962.

[bb3] Bruker (2018). *APEX2* and *SAINT*. Bruker AXS Inc., Madison, Wisconsin, USA.

[bb4] Dolomanov, O. V., Bourhis, L. J., Gildea, R. J., Howard, J. A. K. & Puschmann, H. (2009). *J. Appl. Cryst.* **42**, 339–341.

[bb5] Gammon, J. J., Gessner, V. H., Barker, G. R., Granander, J., Whitwood, A. C., Strohmann, C., O’Brien, P. & Kelly, B. (2010). *J. Am. Chem. Soc.* **132**, 13922–13927. 10.1021/ja1060966 20843035

[bb6] Grabulosa, A. (2011). *P-Stereogenic Ligands in Enantioselective Catalysis.* Cambridge: RSC Publishing, .

[bb7] Groom, C. R., Bruno, I. J., Lightfoot, M. P. & Ward, S. C. (2016). *Acta Cryst.* B**72**, 171–179. 10.1107/S2052520616003954 PMC4822653 27048719

[bb8] Krause, L., Herbst-Irmer, R., Sheldrick, G. M. & Stalke, D. (2015). *J. Appl. Cryst.* **48**, 3–10. 10.1107/S1600576714022985 PMC4453166 26089746

[bb9] Kuzu, M. Y., Schmidt, A. & Strohmann, C. (2024). *Angew. Chem. Int. Ed.* e202319665. 10.1002/anie.202319665 38427610

[bb22] Lide (2005). Editor. *CRC Handbook of Chemistry and Physics, Internet Version*. Boca Raton, FL: CRC Press.

[bb10] McKinnon, J. J., Jayatilaka, D. & Spackman, M. A. (2007). *Chem. Commun.* pp. 3814–3816. 10.1039/b704980c 18217656

[bb11] Monsanto Chemicals (1962). American Patent No. US3053900A

[bb12] Muci, A. R., Campos, K. R. & Evans, D. A. (1995). *J. Am. Chem. Soc.* **117**, 9075–9076.

[bb13] Schillmöller, T., Herbst–Irmer, R. & Stalke, D. (2021). *Adv. Opt. Mater.* **9**, 2001814.

[bb14] Sheldrick, G. M. (2015*a*). *Acta Cryst.* A**71**, 3–8.

[bb15] Sheldrick, G. M. (2015*b*). *Acta Cryst.* C**71**, 3–8.

[bb16] Spackman, M. A. & Jayatilaka, D. (2009). *CrystEngComm*, **11**, 19–32.

[bb17] Spackman, P. R., Turner, M. J., McKinnon, J. J., Wolff, S. K., Grimwood, D. J., Jayatilaka, D. & Spackman, M. A. (2021). *J. Appl. Cryst.* **54**, 1006–1011. 10.1107/S1600576721002910 PMC8202033 34188619

[bb18] Stalke, D. (1998). *Chem. Soc. Rev.* **27**, 171.

[bb19] Tang, W. & Zhang, X. (2003). *Chem. Rev.* **103**, 3029–3070. 10.1021/cr020049i 12914491

[bb20] Verschoor-Kirss, M. J., Hendricks, O., Verschoor, C. M., Conry, R. & Kirss, R. U. (2016). *Inorg. Chim. Acta*, **450**, 30–38.

[bb21] Wolfe, B. & Livinghouse, T. (1998). *J. Am. Chem. Soc.* **120**, 5116–5117.

